# B7-H4: a multifaceted immune checkpoint and oncoprotein in cancer biology and immunotherapy

**DOI:** 10.3389/fimmu.2026.1837397

**Published:** 2026-05-13

**Authors:** Chunyu Zhang, Zhiwei Miao, Jingjing Cao, Xiaoyu Wang, Tongguo Shi

**Affiliations:** 1 Department of Gastroenterology, Zhangjiagang TCM Hospital Affiliated to Nanjing University of Chinese Medicine, Zhangjiagang, China; 2 Jiangsu Institute of Clinical Immunology, The First Affiliated Hospital of Soochow University, Suzhou, China

**Keywords:** B7-H4, biomarker, cancer, cancer immunotherapy, immune

## Abstract

Immunotherapy has become a cornerstone of modern oncology. While immune checkpoint inhibitors have achieved transformative outcomes across multiple cancers, a substantial proportion of patients exhibit primary or acquired resistance, highlighting the need to identify novel immune regulatory pathways. The B7 family member B7-H4 (VTCN1) has emerged as a pivotal co-inhibitory checkpoint that is frequently overexpressed in various solid malignancies. It exerts potent immunosuppressive effects by impairing T-cell function and shaping an immunosuppressive tumor microenvironment. Concurrently, B7-H4 drives tumor-intrinsic oncogenic programs, promoting cell cycle progression, epithelial-mesenchymal transition, stemness, and resistance to therapy. Clinically, elevated B7-H4 levels correlate strongly with advanced cancer stage, metastasis, and poor prognosis, underscoring its dual utility as a prognostic biomarker and a compelling therapeutic target. Consequently, B7-H4-directed therapies, including monoclonal antibodies, bispecific T-cell engagers, antibody-drug conjugates, and chimeric antigen receptor T cells, hold significant potential to improve outcomes for cancer patients.

## 1 Introduction

Immune checkpoints are a class of cell surface receptors and ligands that deliver co-stimulatory or co-inhibitory signals to precisely modulate the activation, differentiation, and effector functions of adaptive immune cells (1–3). Programmed cell death protein 1 (PD-1), its ligand PD-L1 (CD274), and cytotoxic T lymphocyte-associated protein 4 (CTLA-4) are among the best-characterized inhibitory immune checkpoints. They serve as key mediators of T cell exhaustion and apoptosis, thereby suppressing antitumor immunity and enabling tumor immune evasion (4–6). Immune checkpoint blockade targeting these molecules has revolutionized cancer therapy, with anti-PD-L1 and anti-CTLA-4 antibodies having significant survival benefits in select malignancies (7–9). Nevertheless, a substantial proportion of individuals exhibit primary or acquired resistance. In addition, these agents show limited efficacy across many tumor types, underscoring the urgent need for next-generation immunomodulatory strategies capable of reinvigorating antitumor immunity in nonresponder populations (10, 11).

The B7 family comprises a group of structurally related type I transmembrane glycoproteins that are primarily expressed on activated antigen-presenting cells (APCs). However, several members of this family are also found on lymphocytes, other immune cells, and malignant cells (12). These ligands function as critical immunomodulatory molecules by delivering co-stimulatory or co-inhibitory signals that finetune T cell responses in conjunction with T cell receptor engagement via peptide-major histocompatibility complexes (1, 13). While a subset of B7 family members (e.g., B7-1/CD80 and B7-2/CD86) provides activating signals that amplify T cell activation, proliferation, and effector function, the majority, including B7-H1 (PD-L1/CD274), B7-H3 (CD276), B7-H4 (VTCN1/B7x/B7S1), and B7-H5 (VISTA/PD-1H), predominantly exert immunosuppressive effects (14–16). Co-inhibitory B7 ligands dampen immune activation, promote T cell exhaustion, and facilitate immune evasion by engaging their respective receptors on T cells (14, 15). To date, 10 B7 family members have been identified: B7-1 (CD80), B7-2 (CD86), B7-DC (PD-L2/CD273), B7-H1, B7-H2, B7-H3, B7-H4, B7-H5, B7-H6, and B7-H7 (14–16).

B7-H4 is a type I transmembrane glycoprotein first identified in 2003. It functions predominantly as a co-inhibitory immune checkpoint that suppresses T cell-mediated antitumor responses (17, 18). B7-H4 exhibits a highly restricted expression pattern in normal tissues, in contrast to many canonical immune checkpoints that are constitutively expressed on immune cells. According to the Human Protein Atlas, B7-H4 protein expression is highest in normal nasopharyngeal and bronchial epithelium. However, it is largely undetectable in most vital organs, with low levels observed in the placenta, pancreas, and select activated immune subsets. Notably, B7-H4 is overexpressed in a wide array of solid tumors, including ovarian, breast, non-small cell lung, renal, pancreatic, endometrial, and prostate carcinomas (17). Accumulating evidence demonstrates that B7-H4 contributes to immune evasion and tumor-intrinsic processes such as proliferation, metastasis, epithelial-mesenchymal transition (EMT), and resistance to chemotherapy and targeted therapies, collectively driving adverse clinical outcomes (19).

B7-H4 has emerged as a compelling therapeutic target by leveraging its tumor-selective expression and dual roles in immune suppression and oncogenesis. A growing arsenal of B7-H4-directed agents, including monoclonal antibodies (mAbs), bispecific antibodies (BsAbs), and antibody-drug conjugates (ADCs), such as XMT-1660, AZD8205, and SGN-B7H4V, has advanced into preclinical and early-phase clinical trials, yielding promising antitumor activity (20). The present review therefore summarizes recent advances in understanding the biology and therapeutic targeting of B7-H4, with a particular focus on its implications for cancer immunotherapy, and outlines future directions for clinical translation.

## 2 Biological characteristics of B7−H4

### 2.1 Headings

**Figure 1:**
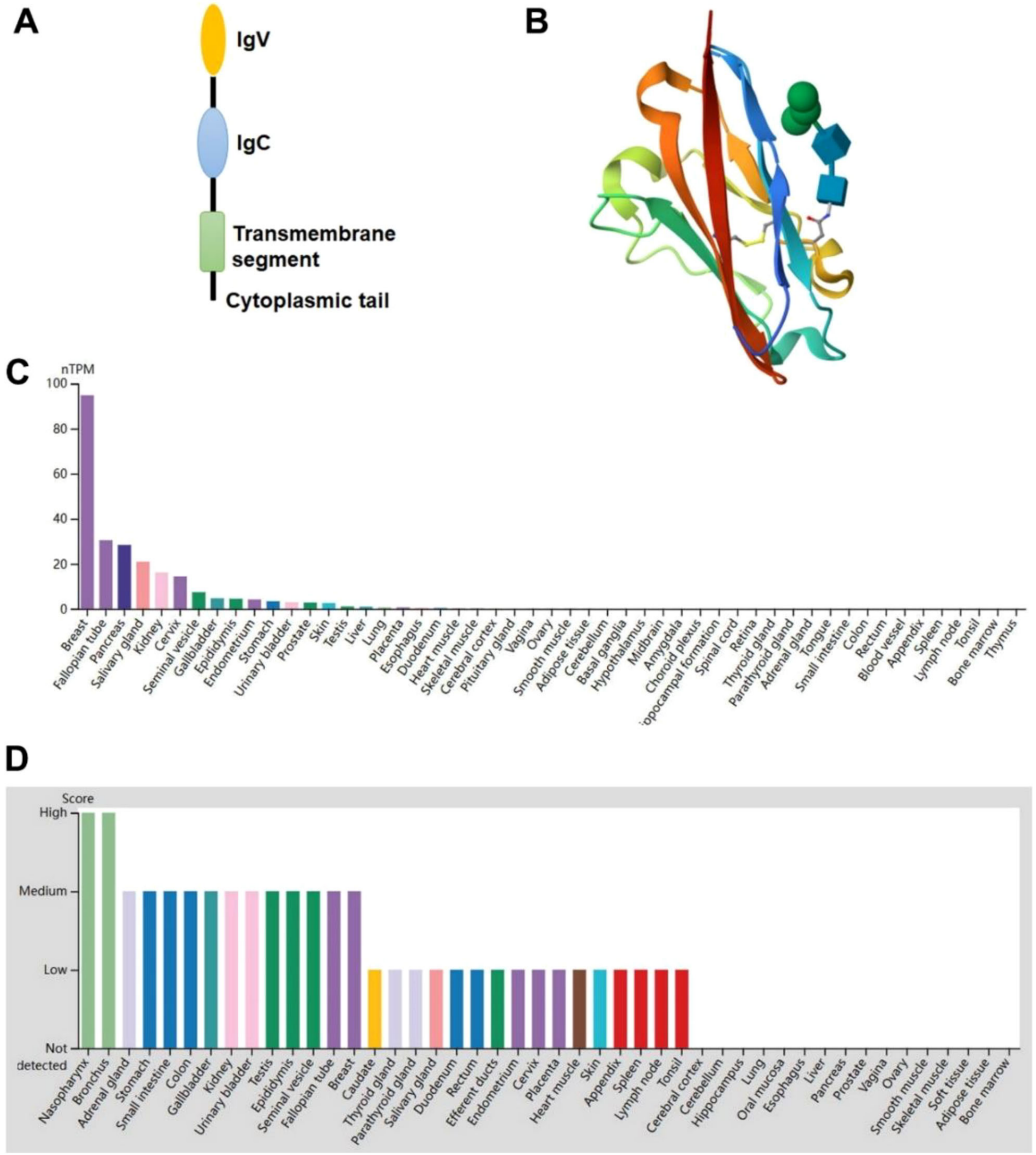
B7-H4 structure and expression. **(A)** Full-length human B7x consists of a receptor-binding IgV domain, IgC domain, stalk region, transmembrane domain, and very short cytoplasmic tail with no signaling motif. **(B)** Crystal structure of human B7-H4 IgV-like domain (PDB: 4GOS, https://www.rcsb.org/). **(C, D)** Human B7-H4 mRNA **(C)** and protein **(D)** expression level across human tissues (https://www.proteinatlas.org).

The human VTCN1 gene that encodes B7-H4 is located on chromosome 1p12–13.1. The full-length B7-H4 protein consists of 282 amino acids and has a canonical type I transmembrane architecture. Specifically, an N-terminal signal peptide is followed by two extracellular immunoglobulin (Ig)-like domains—an N-terminal Ig variable (IgV) and a membrane-proximal Ig constant (IgC) domains—connected via a stalk region to a single-pass transmembrane segment and a notably short cytoplasmic tail that lacks established signaling motifs (**Figures 1A, B**) (21). B7-H4 is evolutionarily conserved across mammalian species, with orthologs identified in non-human primates, canines, and rodents. Human and murine B7-H4 proteins share 87% of amino acid sequence identity, underscoring its functional importance (22).

Beyond its classical role as a membrane-bound immune checkpoint ligand, B7-H4 displays notable subcellular plasticity. In addition to surface expression, it can accumulate in the cytoplasm, translocate to the nucleus via a functional C-terminal nuclear localization signal (NLS), and be released into the extracellular milieu as a soluble isoform (sB7-H4), likely through alternative splicing or proteolytic shedding (23–25). Nuclear B7-H4 has been shown to contribute directly to tumorigenesis by modulating gene expression programs that suppress apoptosis, drive cell cycle progression, and enhance therapeutic resistance. This highlights its dual role as both an immune modulator and a tumor-intrinsic oncoprotein (23).

Transcriptomic and proteomic profiling from large-scale consortia, including the Genotype-Tissue Expression project and The Human Protein Atlas, provides insight into the physiological expression landscape of VTCN1. Integrated analysis reveals that VTCN1 mRNA is most abundantly expressed in placental tissue, with minimal levels detected in breast tissue (**Figure 1C**). In contrast, the high B7-H4 protein expression levels in normal nasopharyngeal and bronchial epithelium indicate potential post-transcriptional regulation or tissue-specific protein stability (**Figure 1D**). This restricted baseline expression in healthy tissues, particularly its near absence in the vital organs, supports the therapeutic rationale for targeting B7-H4 in cancer, where it is frequently overexpressed, while minimizing the risk of on-target, off-tumor toxicity.

Emerging evidence has established that post-translational modifications critically govern B7-H4 function, dictating protein stability, subcellular localization, and immunosuppressive capacity in a disease-specific manner (26–28). N-glycosylation exhibits the most pronounced pathological divergence. Specifically, hypoxia-driven MGAT1 and ST3Gal-I downregulation in tumors generates immature high-mannose glycans, predisposing B7-H4 to endoplasmic reticulum-associated degradation and diminished surface expression (26). Ubiquitination adds a second regulatory layer through linkage-specific fates. Autocrine motility factor receptor (AMFR)-mediated K48-linked ubiquitination targets B7-H4 for proteasomal degradation, whereas deubiquitinases USP10 and USP2a antagonize this process, such that USP10 stabilizes B7-H4 to suppress tumor immunity (27), and USP2a preferentially removes K48/K63-linked chains in epidermal growth factor receptor-mutant lung adenocarcinoma, thereby reducing B7-H4 degradation (29). Palmitoylation provides distinct stabilization in breast cancer, where ZDHHC3-mediated S-palmitoylation at Cys-130 prevents lysosomal sorting and sustains surface B7-H4-mediated immunosuppression (28). These divergent post-translational modification mechanisms, including glycosylation defects enhancing degradation in malignancy versus deubiquitinase/palmitoylation-driven stabilization, provide a mechanistic rationale for context-specific therapeutics that involve promoting B7-H4 degradation in cancer or stabilizing its expression in autoimmune diseases.

## 3 B7-H4 is a promising prognosis and immunotherapy biomarker across malignancies

B7-H4 is aberrantly overexpressed across a wide spectrum of malignancies, including ovarian, non-small cell lung (NSCLC), colorectal (CRC), pancreatic (PaCa), head and neck (HNSCC), bladder (MIBC), prostate (PCa), and cutaneous squamous cell carcinomas, with minimal or absent expression in normal tissues (20), highlighting its pathological significance and potential as a clinical biomarker and therapeutic target. The B7-H4 expression levels in both peripheral blood and tumor tissues are strongly associated with clinicopathological features and patient prognosis.

Elevated sB7-H4 levels in peripheral blood serve as a noninvasive diagnostic and prognostic indicator. High sB7-H4 serum levels in ovarian cancer strongly correlate with advanced disease stage, lymphatic metastasis, suboptimal surgical outcomes, and platinum resistance (24). The sB7-H4 levels in NSCLC are significantly higher than in patients with benign lung disease or healthy volunteers, and demonstrate superior diagnostic accuracy (AUC) compared to conventional tumor markers, such as CEA and CA125 (30). Similarly, serum B7-H4 levels in colorectal cancer (CRC) are elevated relative to those in healthy controls, and are positively associated with deeper tumor invasion, larger tumor mass, and lymph node metastasis, with combined detection of B7-H4 and CEA significantly improving diagnostic sensitivity and specificity (31).

B7-H4 overexpression at the tissue level is consistently linked to aggressive clinicopathological features, including lymph node metastasis, advanced TNM or pT stage, and poor tumor differentiation in ovarian, lung, CRC, and prostate cancers (32–35), as well as larger tumor size and higher stage in cutaneous squamous cell carcinoma (36). B7-H4 also promotes EMT, as evidenced by its positive correlation with mesenchymal markers (e.g., vimentin) and inverse association with epithelial markers (e.g., E-cadherin) in CRC and ovarian cancer (33, 37), implicating it in metastatic progression. Clinically, high B7-H4 expression is predominantly associated with poor overall survival and reduced recurrence-free survival in ovarian cancer (37, 38), MIBC (34), CRC (33), pancreatic adenocarcinoma (PAAD) (39), and prostate cancer (35), with meta-analyses confirming its significant association with increased risk of disease progression in ovarian cancer (38). Notably, B7-H4 in the nonspecific molecular profile (NSMP) molecular subtype of endometrial cancer paradoxically predicts improved survival (32), demonstrating context-dependent prognostic roles. Importantly, while single expression of B7-H3 or B7-H4 shows limited prognostic value, co-deficiency of both molecules predicts better outcomes in pancreatic adenocarcinoma and defines an “immune-hot” tumor phenotype characterized by enhanced CD8^+^ T cell infiltration (39), suggesting synergistic immunosuppressive functions.

B7-H4 exerts potent immunosuppressive effects within the tumor immune microenvironment, consistently showing a negative correlation with CD8^+^ T cell infiltration in HNSCC (40), pancreatic cancer (41), and other solid tumors, thereby contributing to an immune-cold phenotype. In PaCa, concurrent high expression of B7-H4 and PD-L1 identifies a distinct immunosuppressive subtype with reduced CD8^+^ tumor-infiltrating lymphocyte (TIL) numbers (41), whereas B7-H4-positive tumors often display low stromal TIL density and worse outcomes, particularly in ovarian serous carcinoma (37). Paradoxically, high B7-H4 expression in metastatic CRC is associated with increased CD68^+^ macrophage infiltration and poor prognosis (42), indicating complex interactions with myeloid compartments. Moreover, B7-H4 expression positively correlates with cancer stemness markers, including CD24, CD44, CD133, and ALDH1, at protein and/or mRNA levels (37), linking it to therapy-resistant cell populations.

Collectively, these findings suggest that both soluble and tissue forms of B7-H4 may provide significant clinical utility for diagnoses, risk stratification, and prognoses. Its strong association with poor outcomes across most cancers, except in rare molecular contexts, solidifies B7-H4 as a high-priority target for next-generation immuno-oncology strategies, particularly in rational combinations with PD-L1 or B7-H3 blockade.

## 4 Integrative mechanisms of T cell suppression and tumor microenvironment sculpting by B7-H4

**Figure 2:**
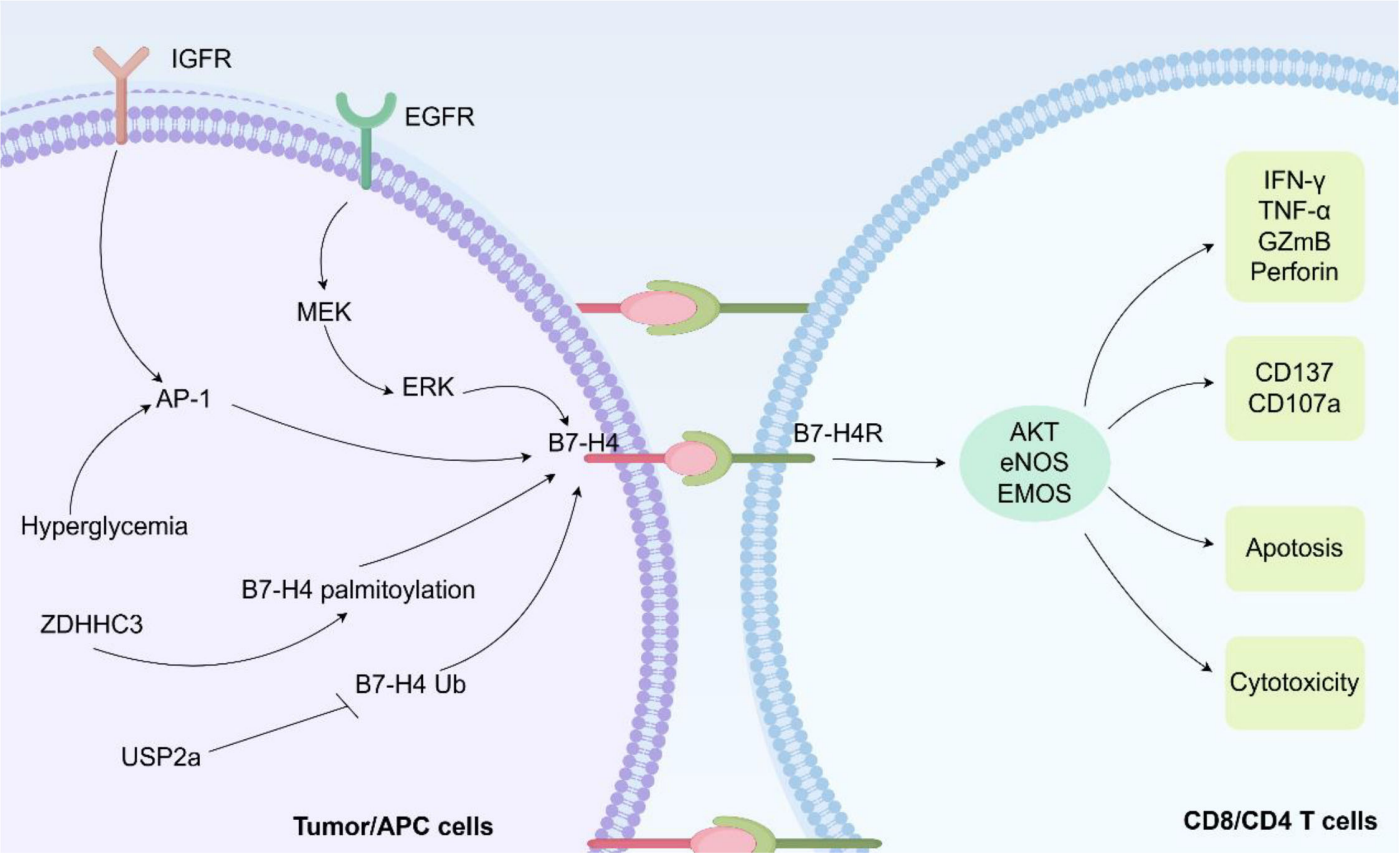
B7-H4 modulates T cell responses in cancer. B7-H4 expression is upregulated in tumor cells and APCs via diverse mechanisms. B7-H4 modulates cytokine production, activation, apoptosis, and cytotoxic function of both CD4^+^ and CD8^+^ T cells.

**Figure 3:**
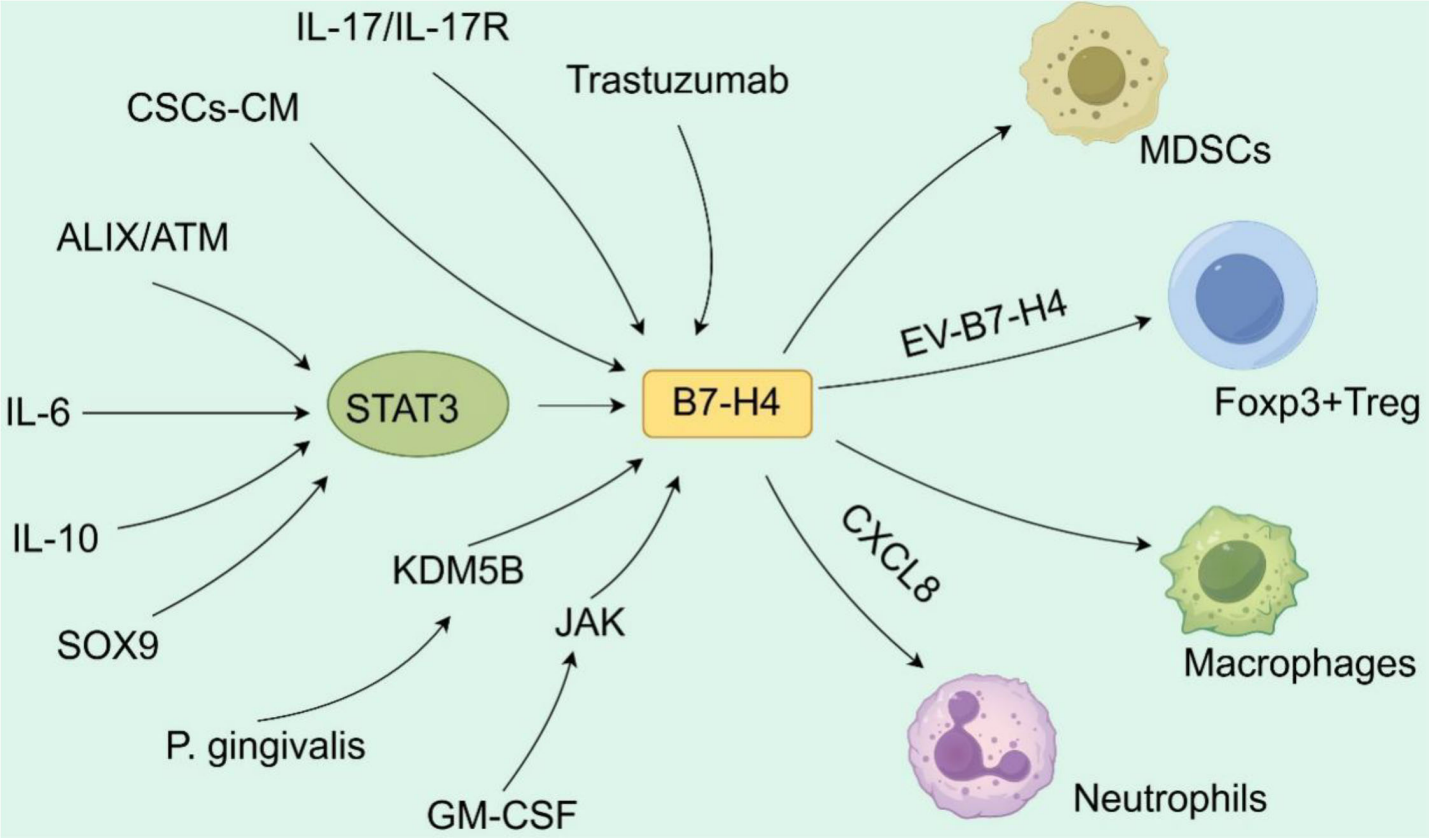
B7-H4 plays a regulatory role in modulating the immunosuppressive tumor microenvironment (TME). B7-H4 expression is upregulated in both tumor cells and APCs through multiple distinct mechanisms. B7-H4 contributes to the establishment and maintenance of an immunosuppressive TME by influencing the recruitment, activation, or function of myeloid-derived suppressor cells (MDSCs), Foxp3+ regulatory T cells (Tregs), macrophages, and neutrophils.

B7-H4 has emerged as a critical negative regulator of T cell-mediated antitumor responses. It functions as a direct T cell checkpoint and as a central hub that integrates diverse signals from the TME to orchestrate a broad immunosuppressive network (14, 43). Its expression is induced by oncogenic signals, inflammatory cytokines, microbial factors, and therapy-induced stress, and modulates diverse immune cell populations to establish a tolerogenic niche that supports tumor progression (44). This section discusses the mechanisms by which B7-H4 regulates tumor-associated T cells (**Figure 2**) and affects the immunosuppressive TME (**Figure 3**), positioning T cell modulation within the broader context of its multifaceted effects on innate and adaptive immunity.

### 4.1 Direct suppression of T cell-mediated antitumor immunity

A unifying theme emerging from multiple preclinical models is that B7-H4, whether expressed by tumor cells or antigen-presenting cells, consistently restrains antitumor immunity by directly impairing the activation, proliferation, effector differentiation, and survival of CD8^+^ T cells within the TME (**Figure 2**) (43, 45).

Analysis of the available evidence reveals three interconnected mechanistic pathways through which B7−H4 drives T cell dysfunction. First, B7−H4 directly suppresses effector functions. It reduces IFN−γ and TNF−α production in CD8^+^ TILs, impairs granzyme B and perforin expressions, and downregulates activation markers, such as CD137 (4−1BB) and CD107a (LAMP−1) (46, 47). Second, these functional defects are linked to intracellular signaling disruption, most consistently to reduced phosphorylation of AKT and endothelial nitric oxide synthase, which compromises T cell survival and metabolic fitness (46). Third, sustained B7−H4 signaling is associated with T cell exhaustion features, including the transcription factor eomesodermin upregulation (48). Although the direct mechanistic link remains to be fully elucidated in the absence of a known receptor, this transcriptional reprogramming likely contributes to the progressive loss of T cell functionality.

Among these mechanisms, AKT signaling suppression and IFN−γ/granzyme B production inhibition represent the most consistently validated pathways, having been demonstrated across multiple tumor models, including ovarian cancer (46), lung cancer (49, 50), bladder cancer (51, 52), and hematologic malignancies (53). In contrast, the relationship between B7−H4 and specific exhaustion-associated transcription factors (e.g., eomesodermin versus T−bet) appears more variable and may depend on the tumor context or antigen exposure duration (48).

Critically, the causal role of B7−H4 in driving these effects is reinforced by loss−of−function studies. Genetic B7−H4 ablation in murine tumor models leads to an increase in tumor−infiltrating CD8^+^ T cell numbers, elevated CD8^+^/CD4^+^ and CD8^+^/regulatory T cell ratios, and restored cytokine production (48, 49, 54, 55). Similarly, antibody−mediated B7−H4 blockade enhances CTL proliferation, cytokine secretion (IFN−γ, IL−2), and resistance to activation−induced apoptosis across multiple cancer types (49, 51, 52, 54, 55). These consistent reversal−of−phenotype data provide strong evidence that the mechanisms described above are not merely correlative but functionally significant.

### 4.2 B7-H4 as a master regulator of immunosuppressive TME

Beyond its direct impact on T cells, B7-H4 exerts complex, context-dependent effects on innate immune lineages, shaping both lymphoid and myeloid compartments to establish a tolerogenic niche (**Figure 3**) (56, 57).

A growing body of evidence shows that B7-H4 exerts complex, context-dependent effects on both lymphoid and myeloid lineages. In particular, host-derived B7-H4 exhibits an apparent functional duality. While it suppresses antitumor T helper 1 (Th1) immunity, it simultaneously constrains the protumor activity of MDSCs. This duality is exemplified in 4T1 murine breast tumors, where loss of host B7-H4 leads to concomitant upregulation of both cytotoxic immune effector genes and MDSC-associated transcripts, suggesting that B7-H4 simultaneously restrains two opposing arms of the antitumor response (58). Rather than viewing this as an irreconcilable paradox, we propose that B7-H4, as a context dependent immune regulator, has a net impact on tumor progression as a result of its role in different immune lineages. The opposing effects on Th1 cells and MDSCs are likely resolved by temporal dynamics, spatial localization, and cellular context, which are factors that determine which functional axis predominates in a given microenvironment.

Tumor- and stroma-derived B7-H4 further reinforces immunosuppression via direct cellular interactions and paracrine signaling. Irradiation in glioblastoma (GBM) enriches B7-H4 in tumor-derived exosomes, which suppress T cell-mediated antitumor immunity both *in vitro* and *in vivo*. Mechanistically, exosomal B7-H4 promotes FoxP3 expression during Th1 differentiation via STAT1 activation, thereby skewing T cell fate toward the regulatory phenotype (59). Similarly, glioma stem-like cells, particularly U251 CD133^+^ populations, secrete factors that potently induce B7-H4 in monocytes. Their interaction with tumor-associated macrophages (TAMs)/microglia drives reciprocal B7-H4 upregulation in both malignant and stromal compartments through IL-6/IL-10 signaling (60, 61). Clinically, B7-H4 expression in glioblastoma correlates strongly with tumor grade and poor prognosis, underscoring its pathophysiological relevance.

In solid tumors such as infiltrating ductal carcinoma (IDC) and gastric cancer (GC), cytokines and growth factors in the TME directly modulate B7-H4 expression on myeloid cells. IL-6 and IL-10 regulate macrophage B7-H4 across polarization states in IDC (62), while GC-derived GM-CSF activates neutrophils via the JAK–STAT3 axis to upregulate B7-H4, a phenotype associated with advanced disease and reduced survival (63). Likewise, tumor-intrinsic B7-H4 in renal cell carcinoma (RCC) promotes CXCL8 secretion, facilitating the recruitment of tumor-associated neutrophils and further amplifying immunosuppression (64).

Transcriptional and epigenetic mechanisms also converge on B7-H4 to enforce immune evasion. SOX9 in dedifferentiated breast cancer drives B7-H4 expression through both direct promoter binding and STAT3 activation, enabling tumor cells to evade immune surveillance (65). IL-17, which is abundant under inflammatory conditions in pancreatic carcinogenesis, induces B7-H4 to be the most significantly upregulated immune checkpoint during early lesion formation. Genetic B7-H4 deletion delays premalignant progression and alleviates TME immunosuppression (66).

Pathogen-driven immune subversion further exploits B7-H4. Porphyromonas gingivalis is a periodontal bacterium that induces B7-H4 and histone demethylase KDM5B in esophageal squamous cell carcinoma, establishing an infection-associated tolerogenic niche. Dual B7-H4 and KDM5B inhibition synergistically restores both antimicrobial and antitumor immunity, revealing a therapeutic vulnerability at the host-pathogen-tumor interface (67).

Importantly, B7-H4 expression can be dynamically induced by cancer therapies themselves. In HER2^+^ breast cancer, trastuzumab increases macrophage infiltration and phagocytic activity, yet simultaneously upregulates B7-H4 on TAMs. This compensatory immunosuppressive response is linked to poorer clinical outcomes. Co-blockade of B7-H4 with trastuzumab overcomes this resistance, enhancing immune cell recruitment and promoting an antitumor macrophage phenotype (68).

Collectively, these findings establish B7−H4 as a central immunity modulator whose immunosuppressive effects are mediated through a core program of AKT−dependent metabolic impairment, effector cytokine inhibition, and exhaustion−associated phenotype promotion in T cells, while simultaneously exerting context-dependent regulatory effects on myeloid lineages. This unified model is supported by convergent evidence from diverse tumor systems. It positions B7-H4 as a T cell checkpoint, and also as a multifaceted immunosuppressive hub that integrates signals from oncogenic pathways, inflammatory mediators, microbial stimuli, and therapeutic interventions to create an immune-resistant TME. Its multifaceted influence on both adaptive and innate immunity arms solidifies its promise as a high-value target for next-generation immunotherapies aimed at dismantling the immunosuppressive TME and reestablishing durable antitumor responses across solid and hematologic malignancies.

## 5 B7-H4 as a multifaceted driver of tumor-intrinsic oncogenic programs

**Figure 4:**
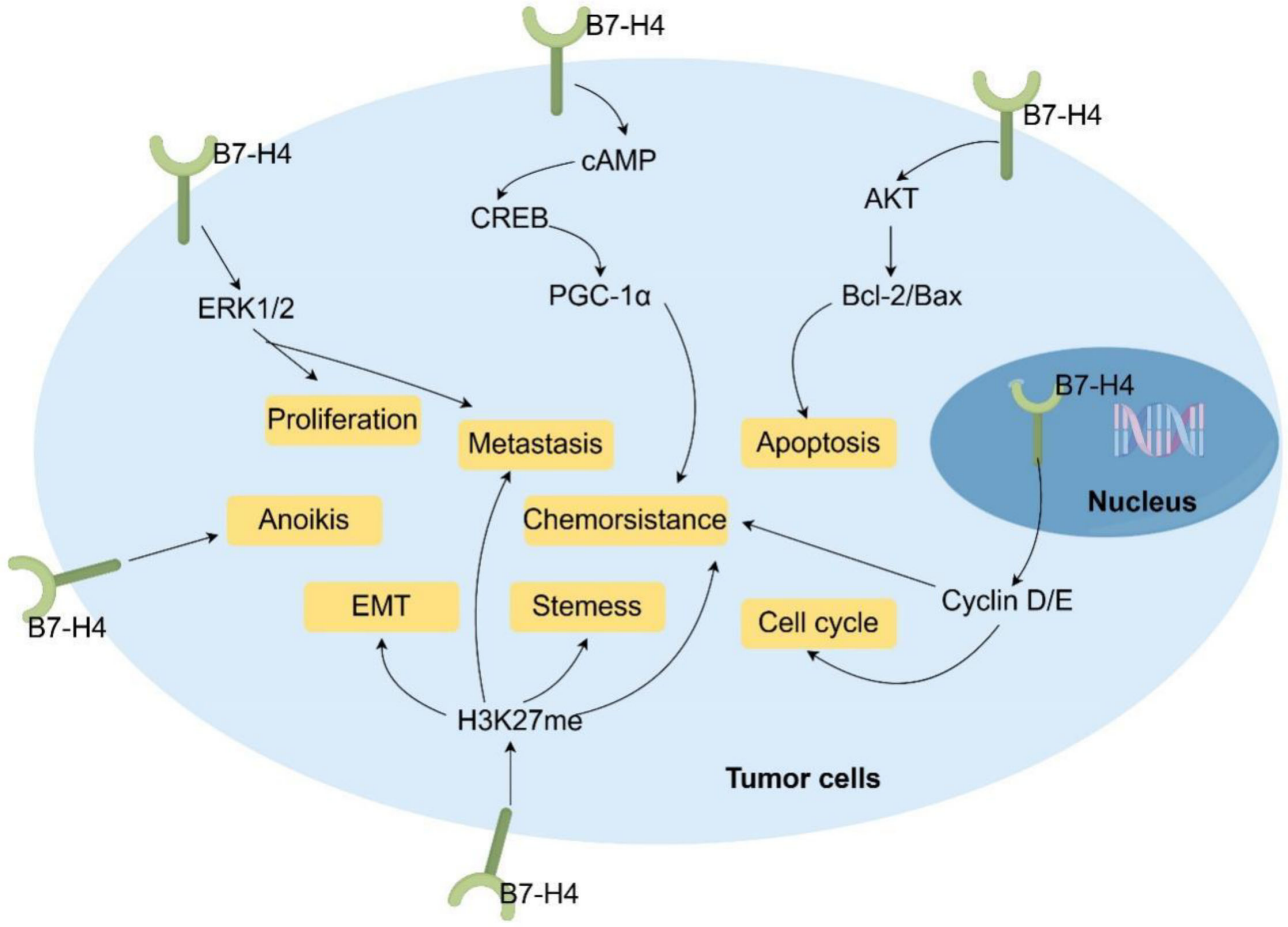
Molecular and cellular mechanisms involving B7-H4-mediated regulation of key oncogenic processes, including proliferation, EMT, metastasis, cell cycle, apoptosis, stemness, chemoresistance and anoikis in cancer.

Beyond its well-established role as an immune checkpoint molecule, B7-H4 exerts important tumor-intrinsic functions that directly regulate cancer cell survival, proliferation, metastasis, stemness, and therapeutic resistance across diverse malignancies (**Figure 4**). Accumulating evidence has demonstrated that B7-H4 modulates core oncogenic signaling pathways and cellular processes independently of immune surveillance (**Figure 4**).

B7-H4 silencing via small interfering RNA in RCC cell lines has been shown to reduce cell viability and sensitize tumor cells to tyrosine kinase inhibitors (TKIs) and mTOR inhibitors (69). This pro-tumorigenic function is critically dependent on the subcellular localization of B7-H4. Nuclear accumulation that is mediated by a functional NLS drives G1/S phase transition through transcriptional upregulation of cyclins D1 and E, and contributes to chemoresistance. Importantly, NLS disruption prevents nuclear translocation and abrogates these oncogenic effects, thereby directly linking the nuclear presence of B7-H4 to its ability to promote cell cycle progression and therapeutic resistance in RCC (23). Similarly, B7-H4 drives proliferation and suppresses apoptosis in lung cancer by modulating the AKT pathway and altering the balance of Bcl-2 family proteins. It downregulates anti-apoptotic Bcl-2 while upregulating pro-apoptotic Bax and caspases-3/8, with its knockdown inducing G0/G1 arrest and impairing *in vivo* tumorigenicity (70).

B7-H4 also governs stress adaptation and mitochondrial homeostasis. B7-H4 downregulation in HeLa cells disrupts mitochondrial function via the cAMP/CREB/PGC-1α axis, thereby enhancing doxorubicin sensitivity (71). Ectopic B7-H4 expression in ovarian cancer increases tumorigenicity in SCID mice and confers resistance to anoikis, facilitating survival during metastatic dissemination (72). Conversely, B7-H4 knockdown in breast cancer cells triggers caspase-dependent apoptosis (72), emphasizing its anti-apoptotic role.

B7-H4 also regulates phenotypic plasticity and aggressiveness. B7-H4 sustains malignant traits in pancreatic cancer, which includes proliferation and migration through ERK1/2 signaling. Its inhibition reverses these phenotypes (73). B7-H4 signaling attenuates AKT activity to modulate cell cycle progression in EBV-positive B-cell lymphoma (74), revealing context-dependent signaling outcomes.

Paradoxically, immune selection pressure in advanced breast cancer drives the “loss” of B7-H4 expression, enabling the escape from B7-H4-directed T cell therapies. However, this downregulation is associated with enhanced EMT, stemness, metastasis, and chemoresistance, which are phenomena that are linked to epigenetic reprogramming, including elevated H3K27me3 levels (75). This highlights a dual role, where B7-H4 supports tumor growth in early stages, and its loss in advanced disease may select for more aggressive, stem-like clones.

Furthermore, B7-H4 is enriched in quiescent brain tumor stem-like cells in gliomas and medulloblastomas, suggesting a function in maintaining a therapy-resistant reservoir (76). EMT-inducing factors [e.g., transforming growth factor-beta 1 (TGF-β1)] and SPC) in lung cancer upregulate B7-H4 via STAT3, while pharmacological inhibition with ethacrynic acid blocks this axis, suppressing metastasis (77).

Collectively, these findings establish B7-H4 as a central regulator of non-immunological oncogenic programs. Its influence spans cell cycle control, apoptosis resistance, mitochondrial function, EMT, stemness, and drug resistance mediated through key pathways, such as AKT, ERK, STAT3, and epigenetic modifiers. This tumor-intrinsic function positions B7-H4 as an immune modulator and a direct driver of cancer progression, with critical implications for therapeutic targeting across tumor stages and contexts.

## 6 Therapeutic strategies targeting B7-H4: from immunomodulation to precision cytotoxicity

B7-H4 has emerged as a compelling target in immuno-oncology due to its pronounced overexpression across a wide spectrum of malignancies, including ovarian, breast, lung, and pancreatic cancers, coupled with minimal or absent expression in most normal tissues (17). This highly restricted expression profile shows that B7-H4 is an ideal candidate for tumor-selective therapeutic intervention, enabling potent antitumor activity while theoretically minimizing on-target, off-tumor toxicities. Consequently, multiple therapeutic modalities are under active preclinical and clinical development, encompassing mAbs, BsAbs, ADCs, and chimeric antigen receptor T (CAR-T) cell therapies.

### 6.1 The mAbs and antibody-derived reagents

The development of B7-H4-targeting mAbs aims to directly block its immunosuppressive signals, thereby restoring T cell function within the TME. Multiple innovative approaches have been used to generate high-affinity anti-B7-H4 antibodies (**Table 1**). An *in vivo* screening platform identified clone 1H3, which is a therapeutic monoclonal antibody that potently suppresses the growth of B7-H4-expressing tumors via multiple antitumor mechanisms. These include enhanced infiltration of T and natural killer cells, reduced MDSC accumulation, decreased levels of vascular endothelial growth factor and TGF-β in the TME, and direct tumor cell killing via antibody-dependent cellular cytotoxicity (78). Similarly, the human monoclonal antibody 5G3 counteracts B7-H4-mediated immunosuppression in lung cancer cells and reduces apoptosis in T cells, preserving their viability and function (79). Beyond full-length antibodies, anti-B7-H4 single-chain variable fragments (scFvs) isolated from patient-derived libraries have been shown to reverse B7-H4-driven T cell inhibition and restore antigen-specific responses, even in complex milieus involving tumor cells, APCs, or TAMs, delaying tumor growth *in vivo* (80). Complementing these efforts, chicken-derived anti-B7-H4 scFvs have also demonstrated efficacy in reversing T cell exhaustion and enhancing activation in human immune cells (81). Furthermore, anti-B7-H4 mAbs have been repurposed as diagnostic tools. For instance, mAb 2H9 radiolabeled with ^89^Zr enables noninvasive, high-fidelity imaging of B7-H4 expression and TAM dynamics in tumors (82). Collectively, these antibodies serve as therapeutic agents and as critical tools for patient stratification and precision immuno-oncology.

**Table 1:** Monoclonal and bispecific antibodies targeting B7-H4.

Cancer type	Animal models	Clone	Mechanisms	Refs.
Colon and breast cancer	B7x/CT26-induced pulmonary metastasis model; 4T1 murine tumor model	B7-H4 mAb 1H3	The mAb 1H3 significantly inhibits the growth of B7-H4-expressing tumors *in vivo* through multiple mechanisms, including enhanced infiltration of T and NK cells, reduced accumulation of MDSCs, decreased levels of VEGF and TGF-β in the tumor microenvironment, and direct tumor cell killing via ADCC	(78)
Lung cancer	/	B7-H4 mAb 5G3	The mAb 5G3 enhances proliferation and reduces apoptosis of T cells	(79)
Ovarian cancer	OVCAR5 xenograft	anti-B7-H4 scFv	The anti-B7-H4 scFv enhances the proliferation, activation, and IFN-γ secretion of antigen-specific T cells.	(80)
/	/	anti-B7-H4 scFvs	Chicken anti-B7-H4 scFvs reverses T-cell exhaustion and enhanced activation	(81)
Prostate cancer	DU145 xenograft TRAMP-C2 tumor	[89Zr]Zr-DFO-2H9	The [89Zr]Zr-DFO-2H9 detects B7−H4 positive tumors and TAM levels in tumors	(82)
Breast cancer	MDA-MB-468 xenograft; PDX	PF-07260437	PF-07260437 enhances the expansion, activation status, and cytotoxic activity of T cells	(83)
Breast cancer	MDA-MB-468 xenograft; hPBMC-transplanted humanized NOG mouse model	B7-H4/CD3-bispecific Fab-scFv antibody	The B7-H4/CD3-bispecific Fab-scFv antibody increases CD8^+^ and granzyme B^+^ CTL infiltration into the tumor.	(84)
Breast cancer	A431 (B7H4) xenograft	B7-H4/CD3-based bispecific antibodies	The bispecific antibody combined with oncolytic virus HSV-2 reveals a synergistic effect in suppressing tumor growth without causing obvious adverse effects.	(85)

### 6.2 BsAbs: redirecting T cells to tumors

BsAbs are engineered antibodies capable of simultaneously recognizing and binding to two distinct targets or epitopes (86). Leveraging this core property of dual-target binding in cancer therapy enables novel mechanisms of action unattainable by traditional monoclonal antibodies, such as physically bridging immune effector cells to tumor cells to facilitate their killing (87, 88). B7-H4×CD3 BsAbs represent a rapidly advancing class of therapeutics designed to physically bridge cytotoxic T lymphocytes (CTLs) with B7-H4-expressing tumor cells, bypassing the need for T cell receptor specificity and converting immunologically “cold” tumors into inflamed microenvironments (**Table 2**). Preclinical studies demonstrate potent antitumor activity, particularly in breast cancer models. PF-07260437 is a B7-H4-targeting CD3 bispecific molecule that enhances CTL-mediated lysis *in vitro* and exhibits synergistic efficacy when combined with endocrine therapy and anti-PD-1 blockade in mouse models (83). Consistently, human peripheral blood mononuclear cells armed with anti–B7-H4/CD3 BsAbs effectively lyse B7-H4^+^ breast cancer lines. Systemic BsAb administration in humanized mouse models trigger rapid and durable tumor regression accompanied by pronounced intratumoral infiltration of CD8^+^ granzyme B^+^ CTLs, with no major adverse effect observed in long-term safety assessments (84). Tang et al. demonstrated that a novel B7-H4- and CD3-targeting bispecific antibody enhances antitumor efficacy in breast cancer when combined with the oncolytic herpes simplex virus 2. This combination regimen induces synergistic suppression of tumor growth and is notably devoid of significant adverse effects (85). These findings emphasize the strong preclinical profile of B7-H4×CD3 bispecific molecules as promising candidates for B7-H4-positive solid tumors.

**Table 2:** ADCs targeting B7-H4 in cancer.

Drug name	Tumor type	Estimated enrollment (N)	Trial phase	Mechanisms	Trial no.	Refs
XMT-1660	Advanced solid tumors	319	Ia/Ib	AF-HPA payload, DAR 6	NCT05377996	(92)
AZD8205	Advanced solid tumors	340	I/IIa	Top1i payload, DAR 8	NCT05123482	(20)
SGN-B7H4V	Advanced solid tumors	30	I	Conjugating MMAE, DAR 4	NCT05194072	(95)

### 6.3 ADCs used in targeted delivery of potent cytotoxic agents

ADCs represent a revolutionary class of targeted cancer therapeutics. They are designed by conjugating the precise targeting specificity of monoclonal antibodies with the potent cytotoxic activity of small-molecule drug payloads (89–91). This innovative strategy aims to selectively eradicate cancer cells while minimizing damage to healthy tissues (89–91). B7-H4-targeted ADCs exploit tumor-selective antigen expression to deliver potent cytotoxic payloads directly to malignant cells, often with bystander effects that overcome heterogeneous antigen expression (20). Three clinical-stage candidates are currently under investigation, each using distinct design strategies that influence their preclinical efficacy, clinical activity, and toxicity profiles (**Table 2**).

The three leading B7-H4 ADCs utilize fundamentally different payload classes and conjugation platforms. XMT-1660 uses the Dolasynthen™ platform to site-specifically conjugate six SN-38-class topoisomerase I inhibitors to a humanized anti-B7-H4 IgG1 at a drug-to-antibody ratio (DAR) of approximately 6 (92). AZD8205 (AstraZeneca) links a distinct camptothecin-derived payload (AZ14170133) via a cleavable linker at a higher DAR of approximately 8 and is designed to induce double-strand DNA breaks and maximize bystander killing (93, 94). In contrast, SGN-B7H4V (Seagen; felmetatug vedotin) uses the vedotin platform, conjugating the microtubule-disrupting agent monomethyl auristatin E (MMAE) at a DAR of approximately 4 via a protease-cleavable mc-vc linker (95). This payload mechanism diversity, including topoisomerase I inhibition for XMT-1660 and AZD8205 versus microtubule disruption for SGN-B7H4V, carries important implications for combination strategies and distinct resistance profiles each agent may encounter.

All three ADCs demonstrated robust antitumor activity in preclinical models. XMT-1660 induced complete tumor regression in multiple breast and ovarian cell line- and patient-derived xenograft models, with superior efficacy observed at DAR 6 compared to DAR 2 or 12 controls. Responses correlated with B7-H4 expression levels, achieving a 75% objective response rate in tumors with a tumor proportion score of 75% or greater (92). AZD8205 regressed by 69% of the level in B7-H4^+^ breast PDX models and exhibited synergistic activity with the poly(ADP-ribose) polymerase-1 inhibitor AZD5305 in BRCA-mutant models, suggesting a potential combination strategy for homologous recombination-deficient tumors (96). SGN-B7H4V achieved a complete regression in breast and ovarian xenografts, with bystander effects enabling efficacy even in tumors characterized by heterogeneous B7-H4 expression (95).

Emerging clinical data reveals both shared features and distinct liabilities among these agents. In the BLUESTAR Phase I/IIa trial (NCT05123482), AZD8205 is being evaluated in patients with advanced solid tumors known to express B7-H4, including breast, ovarian, endometrial, biliary tract, and non-small cell lung cancers. A dose-escalation phase of substudy one enrolled a cohort of 46 patients receiving intravenous doses between 0.8 mg/kg and 3.2 mg/kg once every 3 weeks (Q3W). Preliminary data showed that treatment-emergent adverse events of any grade occurred in 97.8% of the cohort (45 patients). Dose-limiting toxicities were observed at the maximum dose of 3.2 mg/kg, comprising one case each of neutropenia and thrombocytopenia. Two patients (4.3%) discontinued therapy due to treatment-emergent adverse events. A partial response was achieved in 20.9% of the cohort (nine patients) among individuals with ovarian, breast, or endometrial cancer receiving doses of ≥1.6 mg/kg (n=43) (20).

An ongoing phase I trial (NCT05377996) for XMT-1660 is expected to provide efficacy and safety data (92). Similarly, SGN-B7H4V is being evaluated in a phase I trial (NCT05194072) as a monotherapy and in combination with PD-1/PD-L1 inhibitors (96). Although comparative cross-trial interpretation remains premature, the distinct payloads and DAR configurations predict the differences in the therapeutic window that will become clearer as additional data emerge.

The diverse ADC platforms also suggest distinct vulnerability profiles for acquired resistance. For MMAE-based ADCs, such as SGN-B7H4V, resistance may arise through tubulin mutations, upregulation of drug efflux pumps, such as MDR1, or impaired linker processing (97). Potential resistance mechanisms for topoisomerase I inhibitor-based ADCs, such as XMT-1660 and AZD8205, include topoisomerase I mutations, reduced payload accumulation, or alterations in DNA damage repair pathways (98). Notably, the bystander effect common to all three agents may partially mitigate resistance due to heterogeneous antigen expression. It may also increase on-target off-tumor toxicity if payloads diffuse to adjacent normal B7-H4^-^ tissues.

These three ADCs exemplify a precision strategy that merges tumor-selective targeting with potent cytotoxicity, showing particular promise in aggressive, treatment-refractory cancers, such as triple-negative breast cancer and ovarian carcinoma. However, their distinct linker-payload configurations, DAR values, and payload mechanisms are not interchangeable. They exhibit unique efficacy, toxicity, and resistance profiles that will ultimately determine their respective therapeutic niches. Head-to-head comparisons, standardized biomarker assessment, and rationally designed combination strategies, such as pairing AZD8205 with PARP inhibitors or combining SGN-B7H4V with immune checkpoint blockade, will be essential to optimize their clinical deployment and fulfill the therapeutic promise of B7-H4-targeted ADCs.

### 6.4 CAR-T cell therapy: engineered T cell precision

CAR-T cell therapy represents a transformative strategy to reprogram T cells for targeted tumor elimination (99, 100). B7-H4 is an attractive CAR-T target due to its tumor-restricted expression. Preclinical development of a B7-H4-specific CAR demonstrates significant activity. The CAR-T cells exhibit antigen-dependent IFN-γ secretion, specific lysis of B7-H4^+^ targets *in vitro*, and effective control of human ovarian tumor xenografts *in vivo*. However, this efficacy was accompanied by delayed and lethal toxicity traced to on-target, off-tumor reactivity against low B7-H4 levels in select healthy murine tissues (101). This critical finding underscores the importance of safety in targeting B7-H4, despite its relatively restricted expression. Next-generation strategies are being explored to mitigate such risks, including bispecific CAR designs (e.g., targeting B7-H4 in combination with another antigen like mesothelin) or logic-gated CAR architectures that require dual antigen recognition, aiming to preserve antitumor potency while improving the therapeutic window.

In summary, the therapeutic landscape targeting B7-H4 is rich and rapidly evolving, encompassing modalities from immunomodulation (blocking antibodies, bispecific agents) to direct tumor killing (ADCs, CAR-T). While blocking antibodies and bispecific agents seek to reverse immune suppression and engage endogenous immunity, ADCs deliver potent cytotoxic factors with high precision, while CAR-T cells offer the potential for living, targeted therapies. The clinical progress of ADCs and the compelling preclinical data across all platforms validate B7-H4 as a pivotal target. Future success will hinge on defining optimal biomarker strategies (e.g., B7-H4 expression thresholds), managing toxicity profiles (particularly for CAR-T), and exploring their combinations to overcome resistance and maximize patient benefit in B7-H4-positive malignancies.

## 7 Conclusions and perspectives

B7-H4 is a critical inhibitory immune checkpoint molecule within the B7 family that is characterized by its highly tumor-selective overexpression in a wide array of malignancies (17). This distinctive profile is responsible for its dual role as both a key suppressor of antitumor immunity and a tumor-intrinsic oncoprotein. B7-H4 drives tumor progression independently of the immune system by promoting cell cycle progression, resisting apoptosis, inducing EMT, maintaining stemness, and conferring chemotherapy resistance. Clinically, high B7-H4 expression in tissue or serum is strongly associated with advanced stage, metastasis, and poor overall survival in most cancers, confirming its value as a prognostic biomarker. Its tumor-restricted expression has made it an important therapeutic target, leading to the rapid development of diverse agents, such as blocking monoclonal antibodies, bispecific T cell engagers, ADCs, and CAR-T cells. Collectively, these findings highlight the critical role of B7-H4 as a regulator of tumor immune responses and tumorigenesis, positioning it as a potential biomarker and therapeutic target for cancer immunotherapy.

Despite the considerable progress in targeting B7-H4, formidable scientific and translational challenges must be addressed to realize its full therapeutic potential. A fundamental gap remains in the identity of its cognate receptor on T cells, which impedes a comprehensive elucidation of its downstream inhibitory signaling circuitry and the rational structure-guided design of next-generation antagonists. The B7-H4 biology is further complicated by its striking context dependency. For example, while it typically correlates with poor prognosis, B7-H4 expression is associated with improved survival in specific molecular subtypes, such as the NSMP subtype of endometrial cancer (32). Although this association achieved statistical significance in a well-annotated cohort (32), several alternative explanations warrant consideration. It is possible that B7-H4 expression is correlated with an unrecognized protective factor enriched in the NSMP subtype, such as hormone receptor status or specific mutational signatures, thereby introducing the confounding effect. Alternatively, this finding may represent a type I error, highlighting the necessity for validation in larger, independent cohorts using rigorous multivariable adjustment. The paradoxical survival benefit associated with B7-H4 in this context should be interpreted with caution until such confirmatory studies are conducted.

Conversely, immune selection pressure in advanced breast cancer can drive the loss of B7-H4 expression, which is linked to enhanced EMT, stemness, and chemoresistance, revealing a complex and disease stage-dependent role in tumor progression (75). This complexity creates significant hurdles for the straightforward clinical interpretation of B7-H4 as a biomarker. The absence of standardized and validated assays for B7-H4 detection, whether for tissue-based immunohistochemistry (lacking consensus on scoring thresholds and antibody clones) or for quantifying sB7-H4 in serum, severely hinders reproducible patient stratification and cross-trial comparisons from a translational standpoint. Moreover, the mechanisms underpinning potential resistance to B7-H4-directed therapies, whether primary or adaptive, are largely undefined. Perhaps the most critical translational challenge arises from safety concerns, most notably the delayed, lethal on-target/off-tumor toxicity observed in preclinical B7-H4-specific CAR-T cell models. This toxicity is attributed to a low but biologically relevant B7-H4 expression in select healthy tissues. It emphasizes the importance of achieving stringent tumor selectivity. Finally, while early-phase clinical trials, particularly for ADCs, show encouraging signals of activity, the clinical evidence base remains nascent. The definitive efficacy, optimal dosing, and long-term safety profiles of the majority of B7-H4-targeted agents await validation in larger randomized late-phase clinical studies.

Future advancements will necessitate a coordinated, multidisciplinary strategy focused on several key frontiers. First, a deep mechanistic understanding must be pursued by employing cutting-edge techniques in structural biology, proteomics, and functional genomics to unequivocally identify the B7-H4 receptor and map its context-dependent signaling networks across different TMEs. Concurrently, the field must establish harmonized, quantitative biomarker platforms through multi-institutional consortia to define reliable B7-H4 expression thresholds that robustly predict therapeutic response. Regarding therapeutic development, innovation must focus on enhancing specificity and safety. This includes engineering next-generation platforms, such as logic-gated or dual-antigen-targeting CAR-T cells (e.g., requiring co-recognition of B7-H4 and a second tumor-associated antigen) and optimizing ADC designs (e.g., linker-payload combinations) to maximize the therapeutic window. Given the interconnected nature of immune evasion and oncogenic pathways, rationally designed combination regimens will be crucial. Systematic investigation of B7-H4 blockade in combination with PD-1/PD-L1 inhibitors, PARP inhibitors in homologous recombination-deficient cancers, targeted therapies, or conventional chemotherapy holds great promise for overcoming compensatory resistance and inducing more durable responses.

Collectively, the concerted efforts discussed in the present review hold substantial promise for delivering novel, effective therapeutic options to patients who are refractory to current immunotherapies, and for ultimately redefining the standard of care for aggressive B7-H4-positive malignancies.
